# Heart Failure After Right Ventricular Myocardial Infarction

**DOI:** 10.1007/s11897-022-00577-8

**Published:** 2022-10-05

**Authors:** Matthias P. Nägele, Andreas J. Flammer

**Affiliations:** University Heart Center Zurich, University Hospital Zurich, Raemistrasse 100, CH-8091 CardiologyZurich, Switzerland

**Keywords:** Heart failure, Right ventricular myocardial infarction, Right heart failure, Acute coronary syndrome, Inferior STEMI

## Abstract

**Purpose of Review:**

Heart failure (HF) after right ventricular myocardial infarction (RVMI) is common and complicates its clinical course. This review aims to provide a current overview on the characteristic features of RV failure with focus on acute management.

**Recent Findings:**

While HF after RVMI is classically seen after acute proximal right coronary artery occlusion, RV dysfunction may also occur after larger infarctions in the left coronary artery. Because of its different anatomy and physiology, the RV appears to be more resistant to permanent infarction compared to the LV with greater potential for recovery of ischemic myocardium. Hypotension and elevated jugular pressure in the presence of clear lung fields are hallmark signs of RV failure and should prompt confirmation by echocardiography. Management decisions are still mainly based on small studies and extrapolation of findings from LV failure. Early revascularization improves short- and long-term outcomes. Acute management should further focus on optimization of preload and afterload, maintenance of sufficient perfusion pressures, and prompt management of arrhythmias and concomitant LV failure, if present. In case of cardiogenic shock, use of vasopressors and/or inotropes should be considered along with timely use of mechanical circulatory support (MCS) in eligible patients.

**Summary:**

HF after RVMI is still a marker of worse outcome in acute coronary syndrome. Prompt revascularization, careful medical therapy with attention to the special physiology of the RV, and selected use of MCS provide the RV the time it needs to recover from the ischemic insult.

## Introduction

Despite significant progress in the last 20 years, ischemic heart disease remains the single most common cause of death in Europe in both men and women in 2021 [1]. Risk of mortality is highest in the early phase after myocardial infarctions (MI). One of the major complications of AMI is heart failure (HF), occurring both acutely or as a late sequela. In the international GRACE study, incidence of HF on admission was around 16% for both ST-elevation myocardial infarction (STEMI) and non-ST-elevation myocardial infarction (NSTEMI) and 8% for patients with unstable angina [2]. In this registry, the presence of HF as assessed by Killip class was the single most powerful predictor of in-hospital mortality in patients presenting with MI [3]. This has important implications for treatment, as patients with in-hospital HF after ACS are more likely to be medically managed and have longer delays to percutaneous or surgical revascularization [4]. While HF is more commonly seen with left sided MIs (i.e. after occlusion of the left anterior descending artery, LAD, or the circumflex artery, CX) [5], HF due to right ventricular (RV) MI has several unique hemodynamic and electrocardiographic features that need to be considered by treating physicians.

## Pathophysiology

Isolated RVMI is a rare event and only occurs in around 3% of cases [6]. Likewise, in the SHOCK registry on cardiogenic shock complicating acute MI, isolated RV shock was only identified in 3% of patients, whereas left ventricular (LV) failure was the dominant cause of shock (79% of patients) [7]. RVMI is typically seen in the context of inferior STEMI (primarily involving the inferior wall of the LV), where RV involvement can be seen in up to 50% of patients [6]. Interestingly, signs of RV dysfunction are seen in up to 40% of patients presenting with cardiogenic shock due to acute MI [8] and up to one-third of patients with anterior infarcts [9].

**Fig. 1:**
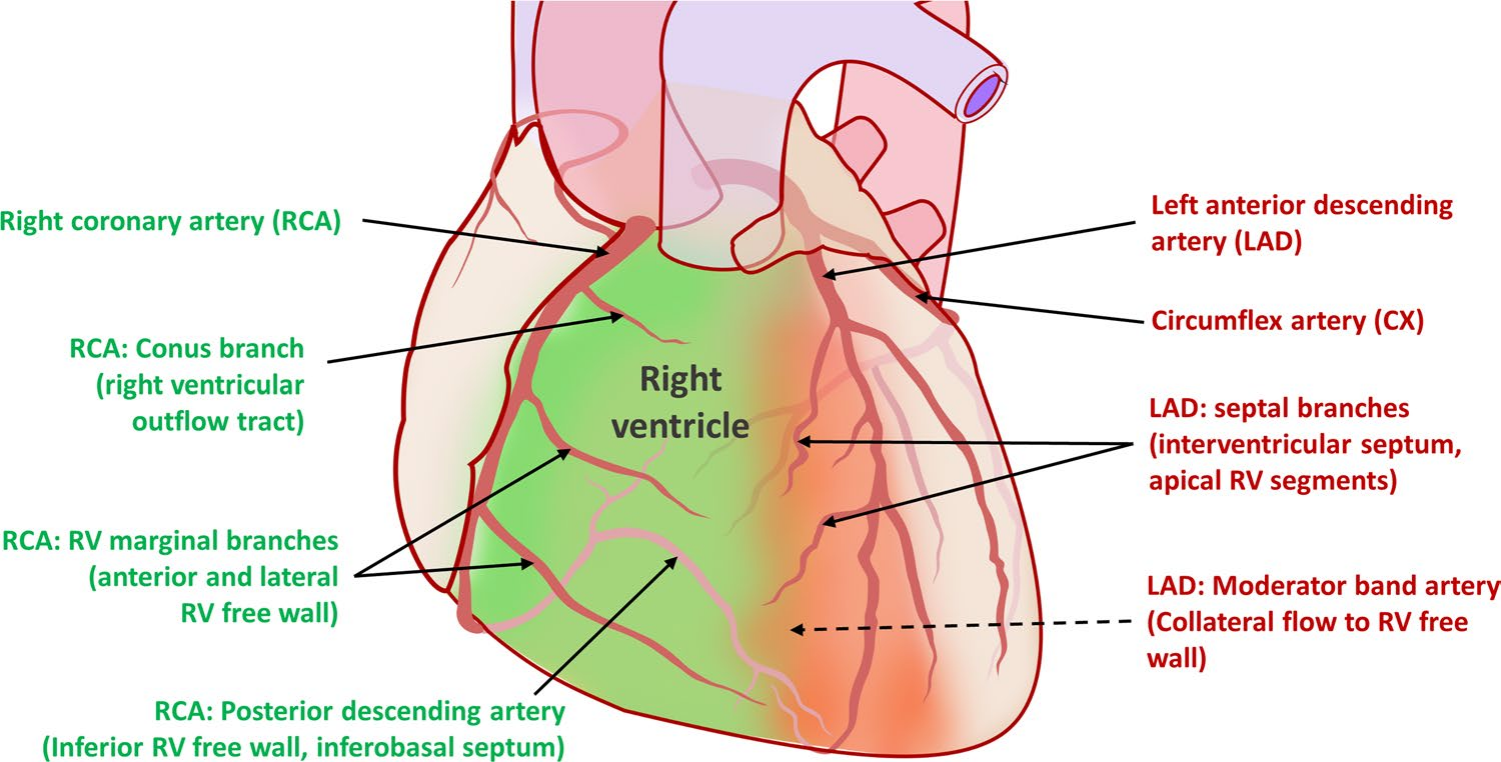
Coronary blood supply to the right ventricle (RV). In the most common variant (right dominance), the majority of blood supply of the free wall is supplied by the right coronary artery (RCA) and its branches (marked in green). The interventricular septum, which contributes significantly to RV function, is mainly dependent on flow from the left anterior descending artery (LAD; marked in red). Considerable variations in anatomy exist. In left dominance, the posterior descending artery is branching from the circumflex artery and thereby originates from the left side. Figure adapted from source image by Patrick J. Lynch and Mikael Häggström, released under Creative Commons Attribution-Share Alike 3.0 Unported license

This is due to the blood supply of the RV (Fig. 1): The main segments of the RV, especially the RV free wall are usually perfused by the right coronary artery (RCA) via RV marginal branches. Thereby, most RVMI are mediated by proximal occlusion of the RCA before the branching sites of RV marginal branches. The LAD supplies the RV apex, anterior interventricular septum, and part of the RV anterior wall adjacent to the septum. In 15–20% of the population, there is a left dominant coronary anatomy. Here, significant portions of the RV free wall are supplied from the left side, usually via the circumflex artery (CX). This can also be the case with chronic total occlusions of the RCA, where RV blood supply can also be entirely dependent on collateral flow from the left coronary system.

**Fig. 2:**
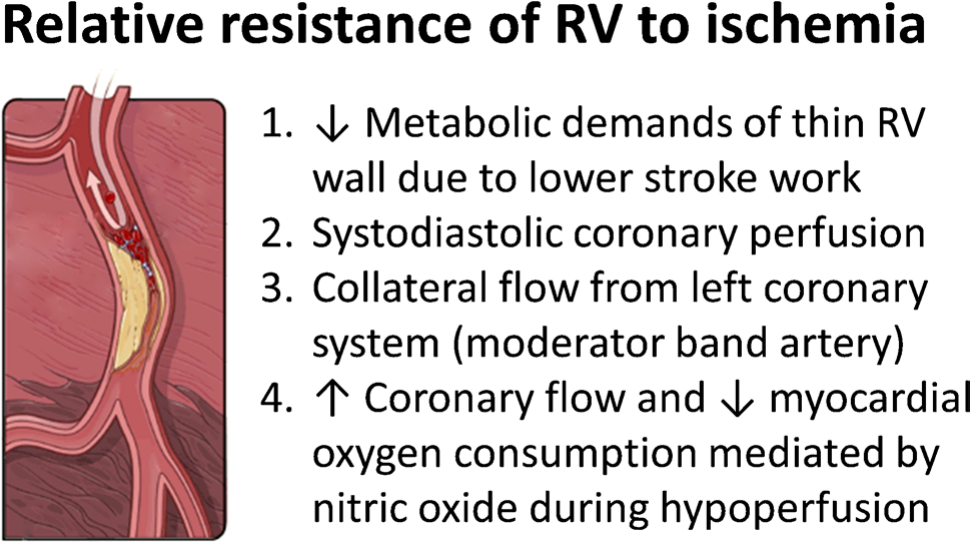
Relative resistance of the right ventricle (RV) to ischemia. Several factors may explain the higher resistance of the right ventricle to ischemic insults. Details explained in the main text

Interestingly, the RV appears to behave differently in response to myocardial ischemia compared to the LV with a higher chance of recovery. Several features suggest that RV dysfunction due to RVMI is more commonly mediated by ischemic stunning of viable myocardium rather than overt infarction with irreversible myocardial necrosis (Fig. 2):

1. Compared to the LV, the RV has lower afterload and lower wall stress in physiologic conditions as it is connected to the low pressure and high-compliance pulmonary circulation. It performs only one-fourth of the stroke work of the LV and has a wall thickness of just one-third of the LV. Resting energy expenditure of the RV is therefore significantly lower, resulting in a larger coronary O_2_ reserve that can be readily mobilized by stressors such as exercise or ischemia [10, 11••, 12].
2. The lower contractile pressures of the RV allow for coronary flow in both systole and diastole as compared to the LV, where most of the flow only occurs during diastole.
3. Autopsies in patients with proximal RCA occlusions showed that the RV is protected from significant infarction by collateral flow from the LAD through the moderator band artery [13]. Massive RV infarctions after proximal RCA occlusion were only seen in patients with concomitant lesions in the LAD territory.
4. The RV can maintain function during moderate coronary hypoperfusion, possibly because coronary blood flow can be increased and myocardial oxygen demand decreased by endogenous nitric oxide release [14].

**Fig. 3:**
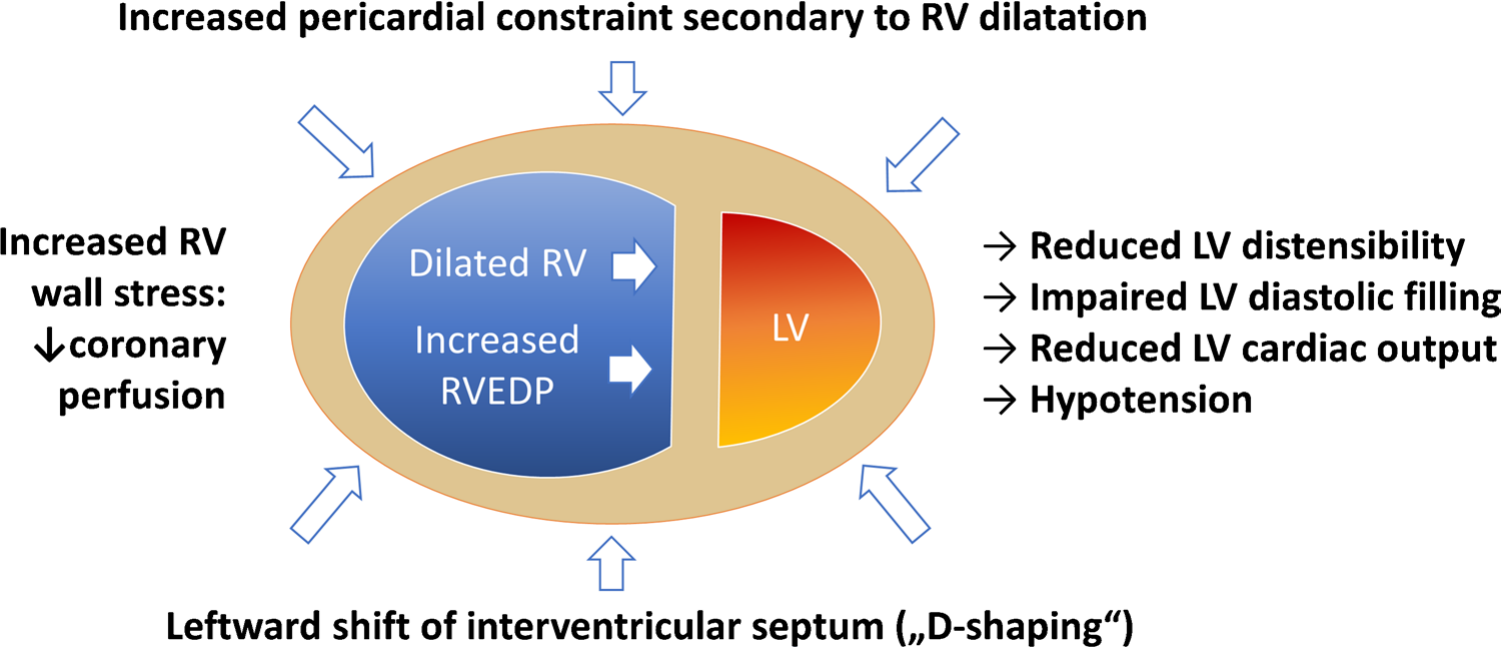
Ventricular interdependence in right ventricular (RV) failure. In RV failure, left ventricular (LV) function is impaired by ventricular interdependence. Increased right ventricular filling pressures (RVEDP) lead to a leftward shift of the interventricular septum (“D-shaping”) because the dilating RV is constrained by the incompliant pericardium. This leads to a change in LV geometry, impairing LV distensibility, preload, and diastolic filling eventually reducing LV cardiac output leading to hypotension and systemic hypoperfusion. These effects are aggravated by increased RV wall stress secondary to RV dilatation which reduces right ventricular coronary perfusion especially in the context of low systemic perfusion pressures

In RVMI with clinically manifest HF, these compensatory measures have failed resulting in significant RV contractile dysfunction. Depressed RV performance due to ischemia decreases the delivery of blood to the LV leading to decreased systemic cardiac output and hypotension. Indeed, hypotension is more common complication in patients with RVMI than anterior MI [15]. Ischemia also impairs diastolic function with increased stiffening and dilatation in late diastole reducing inflow from the right atrium and increasing right atrial and RV filling pressures. A dilated RV with increased filling pressures shifts the interventricular septum toward the underfilled LV (“D-shaping” of the septum in short axis views) which further impairs LV filling. Increased intrapericardial pressure due to rapid RV dilatation also contributes to reduced biventricular compliance and filling due to non-compliance of the pericardium (Fig. 3) [16].

The degree and magnitude of these effects depend greatly on the presence of concomitant LV dysfunction. A significant proportion of RV systolic function is mediated by contraction of the interventricular septum leading to longitudinal shortening of the RV. Hence, RV dysfunction is aggravated when there is prior or concomitant ischemic damage in the LAD territory which supplies the apical and anteroseptal regions of the septum. In larger MIs or in patients with preexisting systolic dysfunction, it is often difficult to attribute the cause of hemodynamic instability specifically to the left or the right ventricle due to their close interplay. In these instances, invasive hemodynamic measurements, such as obtained by right heart catheterization, may be helpful.

## Diagnosis

**Fig. 4:**
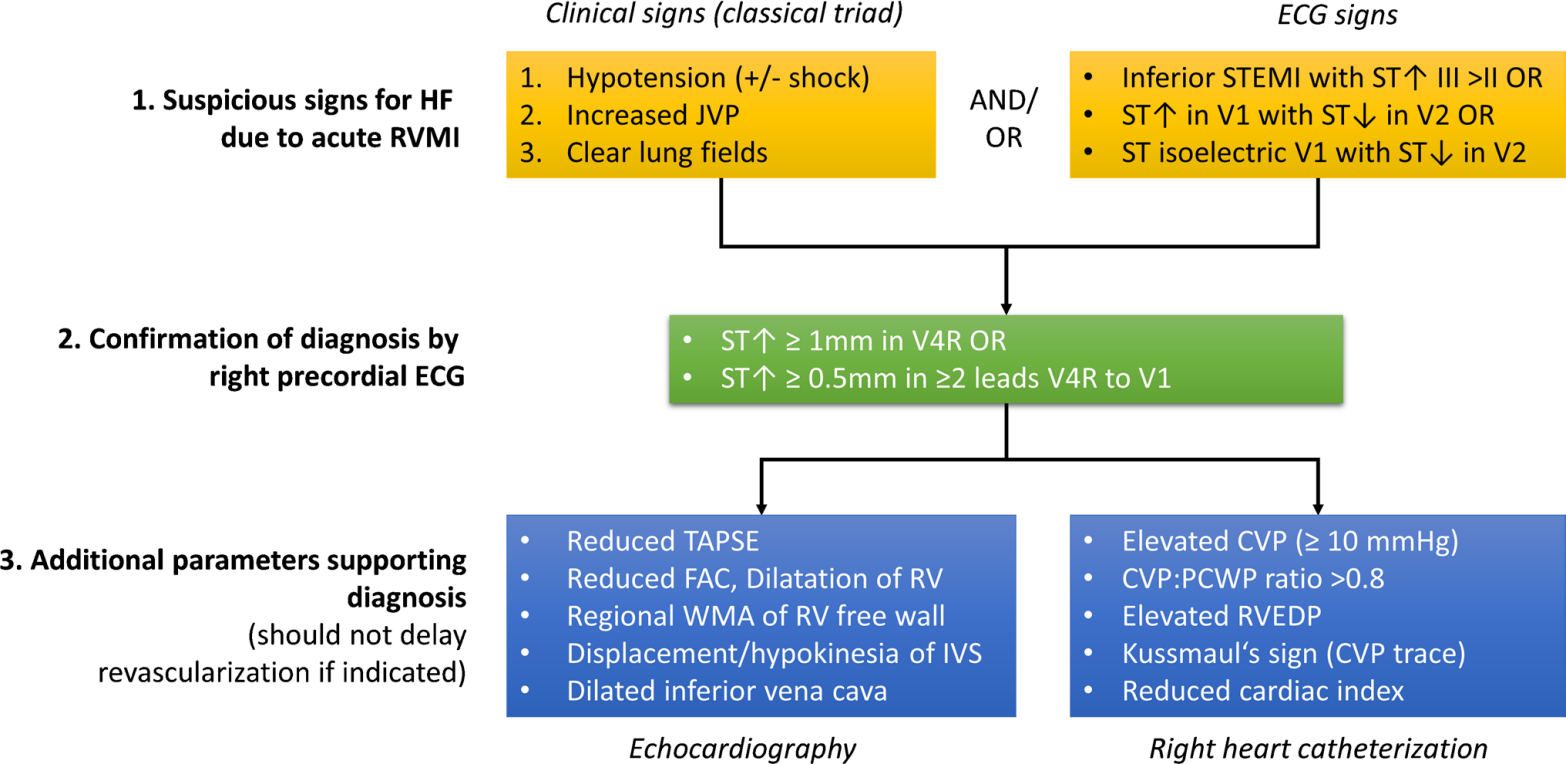
Flow chart for diagnosis of heart failure (HF) in the context of acute right ventricular myocardial infarction (RVMI). Abbreviations: CVP, central venous pressure; FAC, fractional area change of the RV; ECG, electrocardiogram; IVS; interventricular septum; JVP, jugular venous pressure; PCWP, pulmonary capillary wedge pressure; RVEDP, right ventricular end diastolic pressure; ST↑, ST-segment elevation; ST↓, ST-segment depression; STEMI, ST segment elevation myocardial infarction; TAPSE, tricuspid annular plane systolic excursion; WMA, wall motion abnormalities

In patients with symptoms and signs of an ACS (i.e. angina, dyspnoea, ventricular arrhythmias, new regional wall motion abnormalities), RV infarction should be suspected in all patients who present with inferior STEMI in the conventional 12-lead electrocardiogram (ECG). ST-elevation in V1, especially if combined with ST-depression in V2, marked ST depression in V2 combined with an isoelectric ST-segment in V1 or ST elevation in III larger than II (lead III is more rightward facing than lead II) are indicators of RV infarction. Diagnosis is confirmed by obtaining a right precordial ECG. Here, ST-elevation ≥ 1 mm in V4R alone or ≥ 0.5 mm in several leads between V4R and V1 is diagnostic for acute RVMI [17]. The presence of Q waves (QS complexes) in right precordial leads strengthens the diagnosis. A flowchart for diagnosis is shown in Fig. 4.

Clinically, heart failure due to RVMI should be suspected when there is hypotension (± shock) and increased jugular venous pressure (distension of neck veins, positive hepatojugular reflux) in a patient with clear lung fields. Pronounced peripheral oedema, anasarca, ascites, or liver distension are uncommon in the acute phase but represent common signs of decompensation in chronic RV failure.

Echocardiography can be used to further confirm the diagnosis. The complex geometry of the RV makes accurate and reproducible quantification of the RV more difficult in two-dimensional echocardiography. Diagnosis of RV dysfunction should therefore incorporate several different echocardiographic measures of RV function. These include right atrial and RV dimensions (especially in relation to the LV), longitudinal function as measured by tricuspid annular plane systolic excursion (TAPSE) and tricuspid annular systolic velocity, RV fractional area change (FAC), regional wall motion abnormalities of the RV free wall, motion and displacement of the interventricular and interatrial septum, and dilatation of the inferior vena cava. The presence and degree of tricuspid regurgitation and its mechanism are also important, as are evaluation of mechanical infarct complications such as ventricular septal defects or rupture.

Cardiac MRI allows more reliable and detailed quantification of RV structure and function, but is impractical in the context of acutely ill patients with HF associated with acute RVMI and thereby rarely performed.

Invasive measurements using right heart catheterization are not commonly performed in the acute phase and involved with risks (induction of malignant arrhythmias by mechanical irritation of the ischemic myocardium). When they are available, RV failure in the context of RVMI is confirmed by the combination of elevated central venous pressure (CVP ≥ 10 mmHg) and an increased CVP to pulmonary capillary wedge pressure (CVP:PCWP) ratio of > 0.8 [18]. Additional signs are increased diastolic right atrial and right ventricular filling pressures, equalization of LV and RV filling pressures, Kussmaul’s sign in the CVP pressure trace, and a reduced cardiac index.

## Differential Diagnosis

As pericarditis with cardiac tamponade can present similarly, urgent echocardiography should be performed in all patients with inferior MI and hemodynamic instability to rule out this differential diagnosis. Pulmonary embolism is another important differential diagnosis, which can be hard to distinguish from RVMI. Larger pulmonary embolisms can cause significant RV strain due to the acute increase in afterload, with increased troponin and natriuretic peptides and sometimes even ST elevation in V1-V4 and even right-sided leads (i.e. V4R) [19, 20]. Hypoxemia on the other hand can also occur in RVMI, either due to concomitant left heart failure with pulmonary congestion or due to interatrial right-to-left shunting secondary to acutely increased right sided pressures in RVMI [21]. However, inferior ST elevation (as often seen with RVMI due to involvement of the RCA) is uncommon in PE and patients usually report pleuritic instead if anginal pain and may show signs and symptoms of a deep vein thrombosis. Echocardiography is usually not helpful for distinguishing both, as right ventricular dilatation and systolic dysfunction (even regional wall motion abnormalities) can be seen with both RVMI and PE. Normal contraction of the right ventricular apex as opposed to the mid RV free wall (“McConnell’s sign”) is not specific for PE and is seen equally often in patients with RVMI [22, 23]. The presence of wall motion abnormalities in the inferior LV wall as seen with inferior MIs or signs of increased as opposed to normal pulmonary pressure (i.e. increased peak pressure gradient in CW Doppler of the tricuspid regurgitant jet) as seen with massive and submassive PE may be more helpful to distinguish both diagnoses.

## Management

**Fig. 5:**
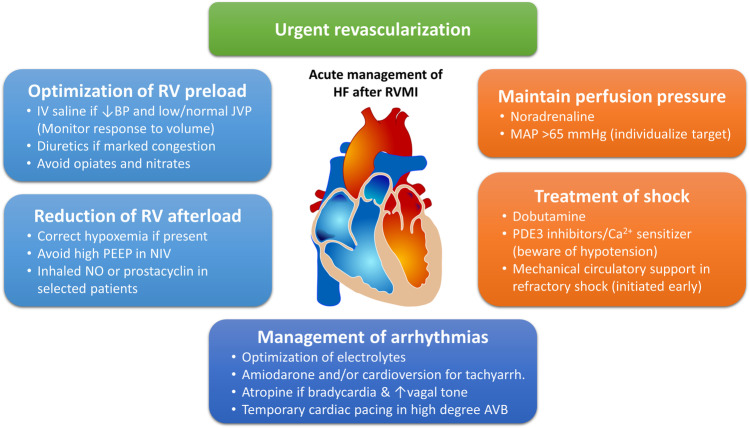
Acute management of heart failure after right ventricular myocardial infarction (Summary). Abbreviations: AVB, atrioventricular block; BP, blood pressure; HF, heart failure; IV, intravenous; JVP, jugular venous pressure; MAP, mean arterial pressure; NIV, non-invasive ventilation; NO, nitric oxide; PDE, phosphodiesterase; PEEP, positive end expiratory pressure; RV, right ventricular; RVMI, right ventricular myocardial infarction; tachyarrh., tachyarrhythmias

Because isolated RVMI with HF is rare and right ventricular HF in the context of inferior STEMI is often considered as a secondary problem, dedicated intervention studies in acute ischemic right-sided HF are rare. Management recommendations for RVMI with HF are therefore mainly based on extrapolations from animal experiments, short-term hemodynamic studies, observational evidence, and expert opinion. Therapy should address the underlying myocardial ischemia, RV preload and afterload, coronary perfusion pressure and end-organ perfusion, and potential tachyarrhythmias and bradyarrhythmias (summarized in Fig. 5).

### Revascularization

In acute RVMI, urgent revascularization, primarily by percutaneous coronary intervention (PCI), is of great importance. In hemodynamic stable patients with STEMI or high-risk NSTEMI, revascularization should not be delayed by further diagnostic tests such as echocardiography and performed as soon as possible (“time is muscle”). Observational evidence indicates that primary PCI is associated with better outcomes in patients with RVMI with both concomitant right-sided HF or cardiogenic shock [24]. Accordingly, successful reperfusion in inferior MI by angioplasty or thrombolysis is associated with rapid recovery of RV function, whereas unsuccessful reperfusion is associated with delayed or no recovery and worse outcomes [25, 26].

### Optimization of Preload

The healthy RV is a compliant structure that can accommodate large volume shifts by increasing contractility in response to preload. However, during ischemia, RV dilatation and diastolic dysfunction make the ventricle susceptible to both volume depletion and overload. Therefore, careful optimization of preload is important in RV failure associated with RVMI. In patients with hypotension and clinical hypoperfusion and low or normal CVP (and normal PCWP if available) and no signs of pulmonary oedema, an intravenous fluid challenge should be considered (i.e. bolus of 250 ml normal saline). Response to fluid resuscitation should be closely monitored using blood pressure and CVP. An increase in CVP > 12 mmHg (or PCWP > 15 mmHg if available) without parallel increase in blood pressure should prompt cessation of volume resuscitation. Further application of volume by increasing RV dilatation and wall stress may worsen RV ischemia and due to pericardial restraint impair cardiac output [16, 27, 28]. In patients with elevated CVP and clinical signs of congestion (i.e. concurrent pulmonary congestion or cardiorenal syndrome), loop diuretics should therefore be considered. In a small observational study, careful loop diuretic therapy but not intravenous fluid application was associated with increased blood pressure after 24 h in patients with acute RVMI and right-sided HF [29]. Drugs that reduce preload such as nitrates or opiates, which are commonly used in AMI, should be avoided in isolated RVMI with HF.

### Reduction of Afterload

**Fig. 6:**
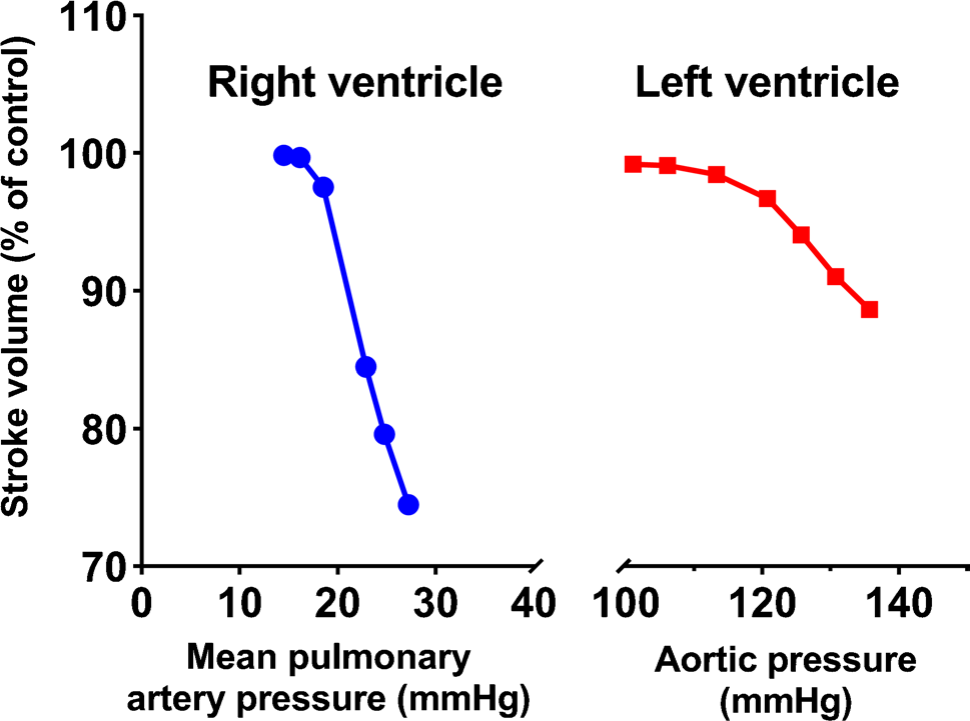
Sensitivity of the right ventricle (RV) to increased afterload. The right ventricle is more sensitive to increased afterload, as seen by a steeper fall in stroke volume in response to increased pulmonary artery pressure in experiments in dogs. The left ventricle on the other side can relatively maintain its stroke volume in response to increased afterload (aortic pressure). Figure reproduced after W. MacNee Am J Respir Crit Care Med. 1994 Sep;150(3):833–52 and Braunwald E. Pathophysiology of heart failure. In: Braunwald E, ed. Heart disease. A textbook of cardiovascular medicine. Philadelphia: Saunders, 1980;453–71. Data based on experiments by Abel et al. J Thorac Cardiovasc Surg. 1967 Dec;54(6):886–94

Due to its thin wall and adaption to a low-pressure system, the RV is more sensitive to acute increases in afterload. An acute increase in pulmonary systolic pressure leads to a greater decline of RV stroke volume than similar increases in aortic pressure affecting LV stroke volume (Fig. 6) [28, 30••]. In isolated HF due to RVMI, afterload should not be targeted specifically by intravenous drug therapies due to their potential of inducing systemic hypotension. Inhaled nitric oxide or inhaled prostacyclin may be considered in selected cases, although they have been primarily studied in patients after cardiac surgery or with severe pulmonary hypertension [31, 32]. A small hemodynamic study in patients with RVMI and cardiogenic shock demonstrated a reduction of right atrial and pulmonary artery pressures and improvement of cardiac index without systemic hypotension after inhalation of nitric oxide [33]. When non-invasive ventilation is necessary, high positive end-expiratory pressure (PEEP) should be avoided, as it can increase RV afterload. This contrasts the effects on the LV, where PEEP has been shown to reduce LV afterload. Hypoxia should also be corrected, as it induces constriction of small intrapulmonary arteries with resulting increase in pulmonary artery pressure and RV afterload. When there is concomitant LV dysfunction, the resulting rise of pulmonary pressure in response to elevated LV filling pressures can further aggravate RV dysfunction by increasing afterload. Here, careful therapies aimed at reducing LV afterload, such as vasodilators, or an intraaortic balloon pump may be helpful in selected cases.

### Vasopressors and Inotropes

There is only very limited evidence to guide the use of vasopressors and inotropes in RVMI with associated HF. Due to their potential of increasing myocardial oxygen consumption and arrhythmias, inotropes are particularly problematic in acute MI. Generally, their use should be limited to patients with end-organ hypoperfusion (i.e. cardiogenic shock) and attempts should be made to wean off these therapies as soon as possible [34••]. However, it should be noted that hypotension may aggravate RV ischemia by further decreasing coronary perfusion. Therefore, maintenance of a sufficient perfusion pressure in right-sided HF due to RVMI is important. After optimization of preload, noradrenaline should be considered a first-line therapy in patients with hypotension and hypoperfusion [35]. Animal studies suggest that noradrenaline increases cardiac output and decreases biventricular filling pressures in RV failure without significantly increasing RV afterload [36]. The targeted mean arterial pressure (MAP) should be individualized based on concomitant elevation of CVP, affected organs, and response to therapy. Generally, a target MAP of 65 mmHg is reasonable [28]. In patients with persisting hypoperfusion despite optimization of preload and afterload and use of noradrenaline, dobutamine should be considered. Dobutamine is more selective for beta-1 adrenoreceptors than adrenaline and has less negative effects on systemic vascular resistance and heart rate [34••]. Since it can decrease systemic vascular resistance, higher doses can cause hypotension since cardiac output cannot be concomitantly increased by the ischemic myocardium, necessitating combined use with noradrenaline. Dopamine is an alternative when dobutamine is not available but is less beta-1 selective, thus increasing heart rate and thereby myocardial oxygen consumption as well as systemic vascular resistance more than dobutamine. Of note, in the SOAP II trial, dopamine increased mortality compared to noradrenaline in the subgroup of patients with cardiogenic shock, mostly due to LV dysfunction [37]. Use of adrenaline should be discouraged as its use is associated with a threefold increased risk of death compared to other inotropes [38] and increased risk of refractory shock compared to noradrenaline in a small randomized trial on cardiogenic shock after MI [39••].

### Phosphodiesterase 3 Inhibitors

Alternatives to dobutamine are PDE3 inhibitors such as milrinone or levosimendan (a combined PDE3 inhibitor and calcium sensitizer) which are also known as ‘inodilators’. Because they decrease systemic vascular resistance through cAMP-mediated vasodilation, their use can be problematic in patients with right-sided HF with predominant hypotension. In a recent randomized trial in cardiogenic shock, milrinone did not show a benefit on clinical outcomes compared to dobutamine [40]. Subgroup analyses did not indicate a benefit in cases with isolated RV failure, although the trial was underpowered to answer this question. While no dedicated endpoint trials exist for RV failure, use of levosimendan in LV failure was associated with worse outcomes compared to placebo in patients with hypotension at baseline in the REVIVE trials [41]. A similar trend (albeit not significant) was also seen in the CHEETAH trial, in patients with LV failure after cardiac surgery with baseline MAP < 60 mmHg, even though no bolus dose was used in this trial [42]. Levosimendan may be more beneficial in patients pre-treated with beta-blockers compared to dobutamine, because at low pre-activation of beta-adrenoreceptors (due to beta-blockade), the calcium sensitizing effect may be more important for inotropy than inhibition of PDE3 [34••, 43]. The problematic long-term effects of PDE3 inhibitors have recently been more appreciated due to their potential to cause maladaptive cardiac remodelling similar to the classical cAMP-dependent inotropes [34••].

### Control of Arrhythmias and Heart Rate

Patients with RVMI have a higher risk for tachyarrhythmias such as atrial fibrillation or ventricular tachycardia/fibrillation compared to anterior MIs [15, 44]. When symptomatic, they should be primarily controlled with electrolyte optimization, amiodarone, and/or cardioversion along with timely revascularization. Beta-blockers should only be used very cautiously due to higher risk of AV block and negative inotropy in RVMI.

RV stroke volume is relatively fixed in RV ischemia, making cardiac output more dependent on heart rate and optimal atrioventricular synchrony. Therefore, bradyarrhythmias are poorly tolerated in RVMI and should be promptly controlled. Patients with inferior STEMI are more sensitive to vagal tone in the first 24 h after infarction, possibly explaining the high incidence of sinus bradycardia at presentation. When there is evidence of increased vagal tone with significant sinus bradycardia or Mobitz type I AV block, atropine should be used first.

High-degree AV block in the context of RVMI with HF is an indication for temporary cardiac pacing [45]. It is often reversible, especially if timely revascularization is achieved. While Mobitz type II block occurs more commonly with anterior STEMI, risk of complete heart block is increased in inferior compared to anterior STEMI and associated with worse outcomes [46]. When there is ongoing atrioventricular dissociation in the context of RV failure, atrioventricular synchronous pacing should be preferred to ventricular pacing alone [45, 47].

### Mechanical Circulatory Support

Use of short-term MCS has increased in cardiogenic shock in recent years. Considering the observation that RV function in RVMI often recovers over time (see chapter prognosis), MCS offers the possibility to bridge critical patients until recovery. Generally, MCS should be considered in patients with RV failure who remain in cardiogenic shock despite optimization of preload and afterload and application of vasopressors and inotropes [48]. Good timing is critical, as late implantation is unlikely to improve outcomes due to complications from multi-organ failure. Patients should be selected according to age, comorbidities, potential for myocardial recovery, and/or eligibility for advanced heart failure therapies such as long-term assist devices or heart transplantation if necessary. Several different devices for RV support are available on the market, although randomized trial evidence for most of them is lacking.

Venoarterial (VA)-extracorporeal membrane oxygenation (ECMO) is a practical and widely available type of short-term MCS that can be used in RV failure [49••]. By draining blood from the right atrium, usually via right femoral vein cannulation, and returning it into the descending aorta via a femoral artery outflow cannula, the RV is effectively bypassed and decompressed while also providing oxygenation in case of concomitant hypoxia.

Retrograde blood flow into the aorta on VA-ECMO also increases LV afterload, which in cases of biventricular failure in the setting of MI can increase RV afterload via backward failure and increased pulmonary artery pressure. Therefore, in cases of biventricular or left-dominant HF with RV involvement, additional device support to allow LV venting may sometimes be necessary such as with percutaneous microaxial flow pumps placed in the left ventricle (e.g. with Impella® pumps, also known as “ECMELLA”). When RV failure predominates despite VA-ECMO, additional jugular venous cannulation (also known as VVA-ECMO) is sometimes sought to improve drainage of the RV using two venous cannulas. Data on this form of cannulation in RV failure in the setting of MI are scarce and should only be tried in selective cases when typical MCS systems have failed.

While use of VA-ECMO in RV-failure is practical, system-specific complications such as differential hypoxia, limb ischemia due to arterial cannulation, bleeding, or LV strain limit its use. Dedicated systems for RV support are available such as the Impella RP® system (Abiomed Inc.) which sucks blood from the inferior vena cava and right atrium and ejects it into the main pulmonary artery using a femorally placed microaxial pump. In a small feasibility study in patients with cardiogenic shock and right-sided HF after cardiac surgery, which also included 5 patients with cardiogenic shock due to MI, invasive haemodynamics improved after Impella RP implantation with overall 30-day survival of 73% in this cohort [50]. However, in a post-market study submitted to the Food and Drug Administration (FDA), 30-day survival after Impella RP® was only 32% [51]. Patients that received the device outside the enrolment criteria of the premarket study, such as patients in cardiogenic shock for longer than 48 h or patients with prior hypoxic or ischemic neurologic events, were much less likely to survive after Impella RP® implantation. This suggests that the device, similar to other MCS devices, should be used early in carefully selected patients before the occurrence of irreversible multi-organ failure.

Other devices for temporary RV support include the TandemHeart® (Livanova Inc.), either using percutaneous arterial and venous cannulation or a dual lumen venous cannula (ProtekDuo®) or surgically implanted devices such the CentriMag® system (Abbott Laboratories). The latter allows support for up to 30 days to bridge patients to transplant, candidacy, recovery, or long-term assist devices.

### Management of Chronic Right-Sided HF After RVMI

Chronic right-sided HF is rare after RVMI. However, when it occurs, it is associated with a worse prognosis. Evidence on disease-modifying treatments in this population are scarce. When there is concomitant systolic LV dysfunction, standard guideline-directed pharmacologic and device therapies should be used first (i.e. quadruple first-line therapy including an ACE inhibitor or sacubitril-valsartan, beta-blocker, mineralocorticoid receptor antagonist, and SGLT2 inhibitor) [48]. Congestion is a common problem in chronic right-sided HF, and patients can be severely symptomatic with peripheral oedema, hepatic congestion, ascites, and progressive renal dysfunction. Hence, loop diuretics are a mainstay of therapy. Typically, intravenous diuretics are used initially to overcome reduced gut absorption in acutely decompensated patients, along with restriction of fluids and sodium. Later, oral diuretics are established under regular monitoring of volume status, kidney function, and electrolytes. Extrapolating evidence from patients with pulmonary arterial hypertension with secondary HF, addition of spironolactone should be considered in right-sided HF, especially to correct diuretic-induced hypokalemia [52]. In advanced right-sided HF with recurrent decompensations, heart transplantation is the only curative therapy as patients are often ineligible for ventricular assist devices.

## Prognosis

The presence of RVMI based on ECG findings (ST elevation in V4R) is associated with worse prognosis in patients with inferior MI as compared to inferior MI without RV involvement [15]. The difference in outcome was not explained by LV infarct size but appeared to be only explained by involvement of the RV. This is mirrored by echocardiographic studies which show that echocardiographic markers of RV dysfunction (i.e. RV FAC, TAPSE, or RV strain) predict outcome in acute MI [53].

However, looking at the culprit vessel, infarction in the LAD or CX territory generally carry a higher risk of HF and mortality in both short (30 days) and long-term follow-up (5 years) compared to RCA infarctions, possibly owing to the higher risk for permanent LV dysfunction and the better potential of the RV to recover [5, 15].

Indeed, when RV function is serially measured after first MI, the majority of patients with reduced RV function show near complete recovery over time [26, 54, 55]. Global RV function even appears to recover quickly in patients who are not revascularized [56–58]. This process is further facilitated by early revascularization [59]. Similar to functional measurements, only a minority of patients show RV scarring as evidenced by late gadolinium enhancement in cardiac MRI at follow-up [9, 55]. However, when RV dysfunction persists after RVMI, long-term survival is reduced [60, 61].

## Conclusions

HF is a serious complication of RVMI which carries a worse short-term prognosis yet good potential for recovery. Early revascularization improves outcomes and should be a main treatment target. Careful optimization of preload and afterload, maintenance of adequate perfusion pressures, good control of arrhythmias, and atrioventricular synchrony as well as timely management of cardiogenic shock with inotropes or MCS as needed can bridge patients until the RV recovers from the ischemic insult.
